# Beyond Blood Pressure: The Role of Flow Parameters and Biomarkers of Hypoperfusion in Cardiogenic Shock

**DOI:** 10.3390/jcm15197492

**Published:** 2026-09-26

**Authors:** Shiun Woei Wong, Yew Woon Chia

**Affiliations:** 1 Department of Cardiology, Tan Tock Seng Hospital, Singapore 308433, Singapore; yew.woon.chia@nhghealth.com.sg; 2 Lee Kong Chian School of Medicine, Nanyang Technological University, 11 Mandalay Road, Singapore 308232, Singapore

**Keywords:** cardiogenic shock, hemodynamic monitoring, flow parameters, cardiac power output, pulmonary artery pulsatility index, mechanical circulatory support, lactate, echocardiography, cardiogenic shock phenotyping, transpulmonary thermodilution

## Abstract

Cardiogenic shock (CS) remains a heterogeneous syndrome with persistently high mortality across acute myocardial infarction, decompensated heart failure, and post-cardiotomy states. Blood pressure, the most commonly used marker of hemodynamic status, is the product of cardiac output and systemic vascular resistance and can be preserved by compensatory vasoconstriction even as flow and tissue perfusion deteriorate. This review examines the evidence for measuring flow-derived hemodynamic parameters and biomarkers of hypoperfusion, rather than relying on pressure alone, to risk-stratify and inform therapy in CS. We propose a practical framework that distinguishes blood pressure targets, measured native flow, device-generated flow, and perfusion biomarkers, and that separates prognostic association from demonstrated treatment-guiding utility, since these are frequently conflated in the existing literature. We summarize data on pulmonary artery catheter-derived cardiac output and cardiac index, cardiac power output and index, the pulmonary artery pulsatility index, transpulmonary thermodilution and other advanced hemodynamic monitoring methods, echocardiographic flow assessment, and perfusion biomarkers including lactate, lactate clearance, and multimarker scores. We review how these parameters are incorporated into the Society for Cardiovascular Angiography and Interventions (SCAI) SHOCK staging and protocols for initiating, titrating, and weaning mechanical circulatory support (Impella, veno-arterial extracorporeal membrane oxygenation), including combined ECPELLA strategies, and we discuss the contemporary randomized trials most relevant to this field, DanGer Shock, ECLS-SHOCK, and ECMO-CS. We also examine how cardiogenic shock etiology and hemodynamic phenotype (e.g., right ventricle-predominant, cardiorenal, cardiometabolic) modify the interpretation and likely generalizability of these parameters. We conclude that integrating flow and perfusion markers with pressure provides a more complete, if not yet fully validated as treatment-guiding, picture of shock severity, while highlighting the continued scarcity of trials that test monitoring strategies themselves rather than the devices they inform.

## 1. Introduction

Cardiogenic shock (CS) is a state of critical end-organ hypoperfusion arising from primary cardiac dysfunction. Despite advances in revascularization, pharmacologic support, and temporary mechanical circulatory support (tMCS), mortality has remained in the range of 30–50% in contemporary series, and the 2025 American College of Cardiology (ACC) concise clinical guidance on CS evaluation and management underscores that individualized, physiology-based assessment, rather than reliance on any single variable, is central to improving outcomes [1].

Blood pressure is the hemodynamic variable most familiar to clinicians and most easily measured at the bedside, but it is a derived quantity: mean arterial pressure (MAP) is, more precisely, related to flow through (MAP − central venous pressure) ≈ cardiac output (CO) × systemic vascular resistance (SVR), since it is the pressure gradient driving flow around the circulation, rather than MAP in isolation, that this relationship describes. Compensatory neurohormonal vasoconstriction can therefore maintain a numerically ‘acceptable’ blood pressure despite a substantial reduction in flow, meaning pressure-guided assessment alone can mask ongoing hypoperfusion or lead to inappropriate escalation or de-escalation of therapies [2]. It is important to state at the outset what this review adds to existing SCAI, ACC, and state-of-the-art documents on CS hemodynamics: rather than treating pressure, flow, and perfusion biomarkers as a sequential list of individually prognostic parameters, as much of the existing literature does, we propose an explicit terminology that separates these constructs (Section 1.1), we distinguish prognostic association from demonstrated treatment-guiding utility for each parameter discussed (summarized in Table 1), and we integrate this across the diagnosis, escalation, monitoring, and MCS-weaning phases of CS care (Section 11). This is intended as a practical, clinically oriented synthesis rather than a systematic review or a new consensus statement. The purpose of this review is to summarize the published evidence supporting the direct and indirect measurement of flow through invasive hemodynamic monitoring, echocardiography, and using biomarkers of tissue perfusion as a complement to, rather than a replacement for, pressure-based assessment in CS, and to describe how these parameters are used across risk stratification and MCS management.

### 1.1. Terminology: Distinguishing Pressure, Flow, Device Flow, and Perfusion

Because the term “flow-based” is used loosely in the literature and, if left undefined, risks conflating distinct physiological and technical constructs, we use it in this review only as an umbrella term and otherwise distinguish explicitly between: (i) blood pressure targets—mean arterial or systolic pressure thresholds used to titrate vasopressors, which do not themselves quantify flow; (ii) measured native systemic blood flow—cardiac output or cardiac index generated by the native heart and quantified invasively (pulmonary artery catheter or transpulmonary thermodilution) or non-invasively (echocardiographic LVOT VTI); (iii) device-generated flow—the flow rate delivered by an Impella pump or the extracorporeal blood flow (EBF) set on a VA-ECMO circuit, which is a machine setting adjusted largely by the operator rather than a direct measurement of native cardiac performance; and (iv) surrogate perfusion biomarkers—lactate, lactate clearance, and venous oxygen saturation, which reflect the net adequacy of oxygen delivery relative to demand rather than flow per se. This distinction is not semantic. As detailed in Section 8, neither DanGer Shock nor ECMO-CS randomized patients to a specific measured-flow or device-flow target: device flow and blood pressure management in both trials were left largely to treating-physician judgment within broad protocol limits, so that extracorporeal blood flow, for example, could reasonably range from approximately 2.5 to 4.5 L/min depending on the clinical scenario. These trials are therefore best described as evidence for flow-augmenting device strategies rather than for flow-guided or flow-targeted monitoring strategies, a distinction we return to explicitly in Section 8. Throughout this review we also try to distinguish, wherever the underlying evidence permits, between a parameter’s demonstrated prognostic association with outcome and its demonstrated ability to improve outcomes when used to guide a specific treatment decision; the latter is a substantially higher evidentiary bar that, for most parameters discussed here, has not yet been met (Table 2).

### 1.2. Literature Search Approach

This is a narrative rather than a systematic review, consistent with the broad, multidomain scope of the topic. We searched PubMed/MEDLINE from January 2000 to March 2026 using combinations of the terms “cardiogenic shock”, “cardiac power output”, “pulmonary artery pulsatility index”, “pulmonary artery catheter”, “transpulmonary thermodilution”, “lactate”, “SCAI SHOCK”, “Impella”, “extracorporeal membrane oxygenation”, “ECPELLA”, and “phenotyping”, supplemented by hand-searching reference lists of recent state-of-the-art reviews and consensus statements. We prioritized randomized controlled trials, contemporary multicenter registries (e.g., the Cardiogenic Shock Working Group and the Critical Care Cardiology Trials Network), and systematic reviews or meta-analyses published within the preceding ten years, while retaining seminal older studies (e.g., the original SHOCK trial registry analyses) that established parameters still in routine use. Non-English-language studies, conference abstracts, and case reports were generally excluded. As with any narrative review, this approach does not guarantee exhaustive or unbiased literature capture, and the final selection inevitably reflects the authors’ judgment of clinical and physiological relevance rather than a reproducible systematic search protocol.

## 2. Why Blood Pressure Alone Is an Incomplete Marker of Shock Severity

The SCAI SHOCK staging system explicitly separates ‘preshock’ (Stage B), defined by hemodynamic instability or reduced cardiac output with preserved systemic perfusion, from overt shock (Stage C and beyond), defined by the presence of hypoperfusion—a distinction that would be impossible using blood pressure alone, since patients in either stage may have similar blood pressures depending on the degree of compensatory vasoconstriction [3]. Registry and consensus data have similarly shown that the biochemical marker most consistently associated with worsening shock stage is not blood pressure but serum lactate, reflecting the fact that tissue hypoperfusion, the physiological hallmark of shock, is a flow-dependent, and not strictly a pressure-dependent, phenomenon [3,4]. Figure 1 illustrates this dissociation schematically: as shock progresses, rising vascular resistance can compensate for falling cardiac output and keep pressure relatively stable, so that pressure declines only late, after flow and perfusion have already been compromised.

A state-of-the-art synthesis of perfusion targets in CS similarly emphasizes that clinicians must draw on physical examination, biochemical markers of end-organ perfusion (renal function, lactate metabolism, venous oxygen saturation) and hemodynamic markers of right ventricular function together, rather than any single blood pressure threshold, because macrocirculatory pressure and microcirculatory perfusion can become dissociated as shock progresses [2]. It bears emphasis that this dissociation can also occur within the flow domain itself: restoration of macrocirculatory flow (e.g., a normalized cardiac index after MCS initiation) does not guarantee normalization of microcirculatory or tissue-level perfusion, since microvascular dysfunction, capillary shunting, and impaired oxygen extraction can persist independently of systemic hemodynamics; sublingual microcirculatory imaging studies in VA-ECMO weaning (Section 7.2) are among the few clinical tools that attempt to assess this directly [5]. This dissociation is the physiological rationale for the flow- and perfusion-based parameters discussed in the remainder of this review.

**Figure 1:**
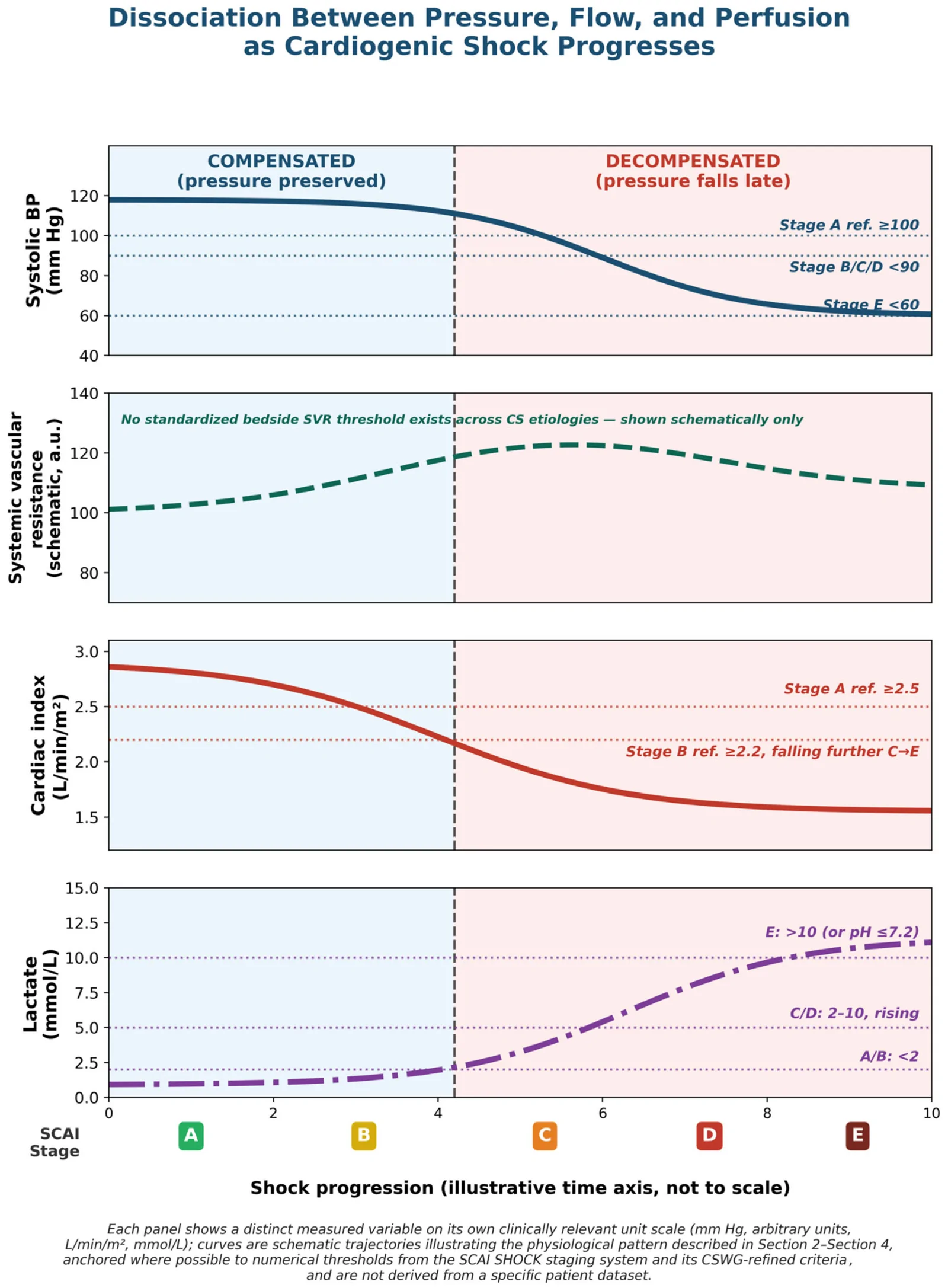
Dissociation between pressure, flow, and perfusion as cardiogenic shock progresses, shown as four separate panels so that each variable is plotted on its own explicit, clinically interpretable unit scale: systolic blood pressure (mm Hg), systemic vascular resistance (schematic, arbitrary units, since no standardized bedside threshold exists across CS etiologies), cardiac index (L/min/m^2^), and lactate (mmol/L). Numerical reference lines are anchored to the SCAI SHOCK staging system and its CSWG-refined criteria [3,6] where applicable. Curves are schematic trajectories illustrating the physiological pattern discussed in Section 2, Section 3 and Section 4 and are not derived from a specific patient dataset.

## 3. Invasive Flow-Derived Hemodynamic Parameters

### 3.1. Cardiac Output, Cardiac Index, and the Pulmonary Artery Catheter

The pulmonary artery catheter (PAC) remains the most established bedside tool for simultaneously measuring cardiac output and the intracardiac filling pressures needed to calculate downstream flow-based indices such as CPO and PAPi; it is not, however, the only bedside method of measuring cardiac output, since transpulmonary thermodilution (Section 3.4) can also quantify flow at the bedside, albeit without direct right heart or pulmonary artery pressure data. Despite decades of controversy about whether PAC placement itself changes outcomes, several large multicenter analyses from the Cardiogenic Shock Working Group registry and the Critical Care Cardiology Trials Network (CCCTN) have found that complete hemodynamic profiling with a PAC, and PAC use more broadly, is associated with lower in-hospital mortality, particularly among patients with more advanced SCAI SHOCK stages [7,8,9]. A systematic review and meta-analysis of PAC use in CS reported a similar association between PAC-guided management and improved survival, although the authors cautioned that this observational association may partly reflect that PAC placement is a marker of more comprehensive, protocolized critical care rather than proof of a direct causal benefit from the catheter itself [10]. A separate meta-analysis of observational studies reached a comparable conclusion regarding the prognostic value of PAC-derived hemodynamic monitoring [11], and data from the pre-MCS era similarly showed changing utilization patterns and outcomes with PAC use in heart failure-related CS [12]. These associations should nonetheless be interpreted cautiously: PAC placement is not randomized in any of these analyses, and residual confounding by indication, unmeasured illness severity, and institutional expertise cannot be excluded, a limitation the authors of several of these analyses explicitly acknowledge [10,11]. These potential benefits must be weighed against the procedural risks of PAC placement itself. Complications are categorized as those related to central venous access (e.g., arterial puncture, pneumothorax), to catheter insertion and manipulation (transient atrial or ventricular arrhythmias, which are common but usually self-limited), and to short- or long-term indwelling presence of the catheter (catheter-related bloodstream infection in approximately 1.3–2.3% of cases, and rare but potentially fatal pulmonary artery perforation or rupture, reported in roughly 0.03–0.2% of catheterizations but carrying a mortality of 30–70% when it occurs) [13]. These risks are generally low in experienced hands but are not negligible, and should be weighed against the incremental hemodynamic information the PAC provides, particularly in less acutely ill patients or where non-invasive or minimally invasive alternatives (Section 3.4) may suffice.

### 3.2. Cardiac Power Output and Cardiac Power Index

Cardiac power output (CPO), calculated as MAP × CO/451, integrates both the pressure and the flow domains of the circulation into a single hydraulic power term expressed in watts. In the original analysis of the SHOCK trial registry, CPO was the strongest hemodynamic correlate of mortality among all measured variables, including cardiac output (CO), cardiac index (CI), and stroke work indices [14]. This finding was later replicated in a broader population of patients with acute cardiac disease, in whom a CPO cutoff below 0.53 W was independently associated with higher in-hospital mortality [15]. A related, slightly more conservative threshold of CPO <0.6 W is also widely used clinically as a practical marker of inadequate cardiac power output; this value was an independent predictor of 30-day mortality in a standardized team-based CS care pathway and has been adopted as a quality metric (attainment of CPO >0.6 W after therapy) by the National Cardiogenic Shock Initiative [16,17]. The two thresholds (0.53 W and 0.6 W) derive from different cohorts and analytic approaches rather than representing a single validated cutoff, which itself illustrates that CPO thresholds should be applied as approximate risk markers rather than precise diagnostic criteria. As the original CPO formula omits right atrial pressure (RAP), subsequent work has proposed an RAP-corrected cardiac power index (CPI_RAP), which improved risk discrimination in a cohort of SCAI Stage B–D CS patients, particularly by identifying patients with congestion that the standard CPI formula did not capture [18]. In patients supported with a percutaneous ventricular assist device (pVAD), a dynamic ‘CPO ratio’, CPO at 24 h divided by early CPO in the first 2 h after implantation, outperformed single time-point CPO and other hemodynamic parameters in predicting 90-day survival, suggesting that the trajectory of flow recovery after MCS initiation may be more informative than any single measurement [19]. CPI has similarly been proposed to characterize the hemodynamic response to Impella support [20]. As with several parameters discussed in this review, it should be emphasized that CPO’s evidence base is predominantly prognostic: it consistently stratifies mortality risk, but no randomized trial has tested whether titrating therapy to a specific CPO target, rather than measuring CPO for risk stratification alone, improves outcomes (Table 2).

### 3.3. Pulmonary Artery Pulsatility Index and Right Ventricular Flow–Pressure Coupling

Right ventricular (RV) failure is common in CS and carries independent prognostic importance [21]. The pulmonary artery pulsatility index (PAPi), calculated as pulmonary artery pulse pressure divided by RAP, was originally developed to identify severe RV dysfunction in inferior myocardial infarction [22] (a PAPi ≤ 0.9 provided 100% sensitivity and 98.3% specificity for severe RV dysfunction and need for RV mechanical support in the original derivation cohort, and this cutoff is the value most consistently cited in subsequent validation and clinical-application studies, though optimal thresholds have varied from approximately 0.9 to 2.0 depending on the population and clinical context studied [22,23]) and has since been validated as a predictor of mortality and need for RV mechanical support across CS, advanced heart failure, and left ventricular assist device candidacy evaluation [23]. Analyses of the SHOCK trial registry demonstrated that RV dysfunction, captured hemodynamically through indices such as PAPi, identifies a distinct high-risk phenotype within CS [24], and data from the ESCAPE trial cohort showed that lower PAPi values independently predicted adverse outcomes in advanced heart failure [25]. As with left-sided cardiac power, PAPi reflects the coupling of right heart flow generation to right heart filling pressure rather than pressure in isolation, reinforcing the broader theme that flow–pressure relationships, and not pressure alone, carry prognostic weight.

### 3.4. Other Methods of Advanced Hemodynamic Monitoring

Because this review argues for looking beyond blood pressure, it is important to situate the PAC among other available methods of measuring flow, even though the PAC remains the dominant tool in the CS literature owing to its unique ability to also sample mixed venous oxygen saturation and pulmonary pressures. Transpulmonary thermodilution (e.g., the PiCCO system) combines an intermittent thermodilution-derived cardiac output measurement with continuous pulse-contour analysis, and additionally provides the extravascular lung water index (EVLWI) and the pulmonary vascular permeability index, which can help distinguish hydrostatic from permeability-type pulmonary edema, a distinction not available from PAC-derived wedge pressure alone. Small comparative studies in CS populations have found close agreement between transpulmonary and pulmonary-artery thermodilution-derived cardiac output and cardiac function indices, including in patients supported with intra-aortic balloon pumps, suggesting the two techniques are broadly interchangeable for flow measurement in this setting [26,27]. Transpulmonary thermodilution requires only central venous and arterial (rather than pulmonary arterial) access, which may make it an attractive, less invasive option earlier in the shock course. However, its validation in CS remains limited to small single-center cohorts, and, notably, its performance has not been established in patients supported with Impella or VA-ECMO, in whom continuous, non-pulsatile or partially pulsatile flow can distort both pulse-contour analysis and, potentially, thermodilution curves themselves. It is therefore most reasonably positioned as a monitoring option in SCAI Stage A–C patients who have not yet required temporary MCS, rather than as a validated substitute for PAC- or device-derived flow data once MCS is initiated. Fully non-invasive techniques (bioreactance, pulse-wave analysis, and finger-cuff volume-clamp methods) have been validated primarily in general critical care and perioperative populations rather than in CS specifically, and their agreement with thermodilution-based cardiac output narrows as hemodynamic instability, vasopressor dose, and peripheral vasoconstriction increase—precisely the conditions present in CS; they are therefore best regarded as trend-monitoring rather than absolute-value tools in this population.

## 4. SCAI SHOCK Staging: A Framework Linking Hemodynamics, Perfusion, and Biomarkers

The 2022 update to the Society for Cardiovascular Angiography and Interventions (SCAI) SHOCK staging system formalizes many of the distinctions discussed above by explicitly separating patients with abnormal hemodynamics but preserved perfusion (Stage B, ‘preshock’) from those with overt hypoperfusion (Stage C onwards) [3]. The consensus document highlights serum lactate as a key discriminator of hypoperfusion across stages, while noting that lactate can be dissociated from hemodynamics in certain populations (e.g., chronic heart failure) and that causes of hyperlactatemia other than shock, i.e., Type B hyperlactatemia, must be considered [3]. Figure 2 summarizes, in schematic form, how flow-based hemodynamics, perfusion biomarkers, and typical MCS considerations broadly track across the five SCAI SHOCK stages, synthesizing the parameters discussed throughout this review; the specific values shown are illustrative rather than fixed cutoffs, since individual thresholds vary by CS etiology and clinical context [2,3]. As discussed further in Section 10, the pace and pattern with which patients move through these stages, and the parameter thresholds that best identify them, are not uniform across CS etiologies and hemodynamic phenotypes. This staging framework has since been used as an outcome discriminator in registries and clinical trial populations, and ongoing validation work continues to refine how physical examination, biochemical, and hemodynamic domains should be weighted and operationalized in practice [3].

## 5. Biomarkers of Hypoperfusion and Their Relationship to Flow

### 5.1. Lactate and Lactate Clearance

Serum lactate is the most widely used point-of-care biomarker of tissue hypoperfusion in CS and a component of most contemporary CS risk scores. In a substudy of the IABP-SHOCK II trial and registry, arterial lactate measured 8 h after hospital admission was a better predictor of 30-day mortality than either the baseline lactate value or lactate clearance calculated between the two time points, suggesting that, unlike in septic shock, the trajectory of lactate change may add less prognostic information than a well-timed single measurement in CS [28]; of note, CS in the IABP-SHOCK II trial and registry was defined clinically by hypotension and end-organ hypoperfusion criteria rather than by a lactate threshold, so lactate was measured as a prognostic biomarker after shock had already been diagnosed rather than serving as a diagnostic criterion itself. A lactate value of 3.1 mmol/L at 8 h showed the best discrimination for early prognosis in this cohort, and a lactate clearance below 3.45%/h between the two time points was independently associated with time to death [28]. This finding has prompted renewed interest in formalizing the timing of lactate measurement in CS care pathways, exemplified by the SCAI ‘Door-to-Lactate Clearance’ initiative, which proposes standardized early and serial lactate assessment as a quality metric in CS management [29]. Lactate elevation in CS is, however, not specific to hypoperfusion. Adrenergic stimulation, whether endogenous or from exogenous catecholamine infusions, can increase aerobic glycolysis and lactate production independent of tissue oxygen delivery, and impaired hepatic lactate clearance, common in CS with hepatic congestion or hypoperfusion, can elevate lactate through reduced clearance rather than increased production. The SCAI SHOCK consensus document explicitly recognizes that hyperlactatemia can arise from causes other than shock (so-called Type B hyperlactatemia), including metformin use, seizures, and certain malignancies, and that distinguishing these requires clinical judgment rather than a fixed threshold [3]. An isolated lactate value should therefore be interpreted alongside the broader hemodynamic and clinical picture rather than in isolation.

### 5.2. Composite and Multimarker Scores

As no single biomarker fully captures the severity of shock, composite scores combining lactate with other biomarkers have been developed. The CLIP score integrates cystatin C, lactate, interleukin-6, and N-terminal pro-B-type natriuretic peptide and was shown to improve mortality risk stratification in CS complicating acute myocardial infarction compared with individual markers alone [30]. Central and mixed venous oxygen saturation (ScvO_2_, SvO_2_), reflecting the balance between oxygen delivery and consumption, provide complementary information to lactate and are frequently measured alongside it when a central or pulmonary artery catheter is already in place, although, like lactate, their prognostic value has been demonstrated more consistently than their value in guiding specific treatment thresholds. Separately, an analysis from the CCCTN Registry demonstrated a continuum of physiological derangements from preshock to classic CS, reinforcing that hypoperfusion markers, not pressure alone, track with this continuum and outcomes [31]. Acid–base status has likewise been shown to add prognostic information: the combination of acidemia and lactic acidosis was independently associated with outcomes in CS beyond shock severity classification alone [4].

## 6. Echocardiographic and Non-Invasive Flow Assessment

Echocardiography provides a non-invasive, repeatable window into flow that complements invasive hemodynamics, particularly the left ventricular outflow tract velocity–time integral (LVOT VTI) as a measurement of stroke volume. In studies of MCS weaning discussed below, LVOT VTI and related indices (fractional shortening, corrected left ventricular ejection time) were consistently among the strongest echocardiographic predictors of successful device removal, more so than ejection fraction alone [32,33,34]. A recognized limitation is that continuous-flow devices such as Impella and veno-arterial extracorporeal membrane oxygenation (VA-ECMO) can generate artefacts that interfere with Doppler-based measurements, and severe left ventricular dysfunction can render simple ejection fraction assessment insensitive to genuine myocardial recovery, both of which argue for combining echocardiographic flow assessment with invasive hemodynamic and biomarker data rather than relying on imaging alone [33,35].

## 7. Flow-Guided Selection, Titration, and Weaning of Mechanical Circulatory Support

### 7.1. Physiological Distinctions Between Impella and VA-ECMO

Before considering flow-guided titration and weaning, it is necessary to distinguish the physiology of the two devices discussed in this section, since “flow” means something different for each. Impella is an intracardiac microaxial pump that draws blood directly from the left ventricle and ejects it into the ascending aorta, thereby actively unloading the left ventricle (reducing LV end-diastolic pressure and wall stress) while simultaneously augmenting forward systemic flow; it does not provide gas exchange, so oxygenation remains dependent on native lung function. VA-ECMO, by contrast, drains venous blood, oxygenates it extracorporeally, and returns it under pressure to the arterial system (typically peripherally, via the femoral artery), providing both substantially higher extracorporeal flow rates (commonly up to 4–6 L/min) and complete gas exchange; however, because retrograde arterial return increases LV afterload, peripheral VA-ECMO can paradoxically increase LV wall stress, worsen pulmonary edema, and, in the absence of an explicit unloading strategy, impede myocardial recovery. This is the physiological rationale for combined-device (“ECPELLA” or “ECMELLA”) strategies, in which a percutaneous LV assist device is added specifically to unload the left ventricle during VA-ECMO support. Observational data and meta-analyses of ECPELLA versus VA-ECMO alone have shown inconsistent effects on early mortality but a fairly consistent signal of increased bleeding, hemolysis, limb ischemia, and need for renal replacement therapy with the combined approach, reflecting the added vascular access and hemolytic burden of a second device; a formal survival benefit for ECPELLA over VA-ECMO alone has not been established in randomized trials [36]. In discussing “flow parameters” below, we therefore distinguish device-generated flow (the Impella or VA-ECMO circuit flow rate, set largely by operator or protocol) from native cardiac output/index (a physiological measurement) and from total systemic flow (the sum of native and device flow, relevant specifically to VA-ECMO), since these are not interchangeable quantities even though all may loosely be described as “flow” in the clinical literature.

### 7.2. Impella

A 2024 review of Impella use in CS and high-risk percutaneous coronary intervention summarizes device selection, escalation, and weaning practice, noting that despite widespread adoption, randomized evidence specifically evaluating Impella has, until recently, been limited [37]. CPO and its indexed variants have been proposed specifically as flow-based metrics to characterize hemodynamic response to Impella support and to help weaning decisions [20,38]. Weaning protocols generally require a composite of hemodynamic (mean arterial pressure, pulsatility), metabolic (lactate), and imaging criteria to be met before Impella support is progressively reduced, rather than any single threshold [39].

### 7.3. Veno-Arterial Extracorporeal Membrane Oxygenation

Because VA-ECMO flow is controlled by the extracorporeal circuit rather than generated by the native heart, weaning requires demonstrating that the heart can independently generate adequate flow once circuit support is reduced. A retrospective study using a standardized flow-reduction protocol found that the ratio of corrected left ventricular ejection time to pulmonary artery wedge pressure (LVETc/PAWP) was an independent predictor of successful weaning, outperforming blood pressure and ECMO flow rate, which did not differ between successfully and unsuccessfully weaned groups [32]. A systematic review that pooled biomarker, hemodynamic, and echocardiographic predictors across the literature similarly concluded that multiparametric assessment was needed, since no isolated variable reliably predicted weaning success [33]. This conclusion has been reaffirmed in more recent systematic reviews and meta-analyses, which again identified LVEF, LVOT VTI, and admission lactate as the most consistent predictors, while noting substantial heterogeneity and generally low-to-moderate study quality across the available evidence [34]. Earlier work from the same general field established some of the founding predictors of ECMO weaning failure, including hemodynamic and clinical variables assessed prior to explantation [40], and comparative echocardiographic-versus-hemodynamic prediction models have since been developed and validated in independent cohorts [35]. Beyond macrocirculatory and echocardiographic parameters, sublingual microcirculatory imaging has been explored as a marker of successful VA-ECMO weaning, on the rationale that restoration of microvascular flow, rather than macrocirculatory flow alone, may better reflect tissue perfusion and true readiness for decannulation, although this remains an investigational rather than standard-of-care tool [5]. As discussed in Section 8, the neutral results of the ECLS-SHOCK trial similarly underscore that device flow augmentation by VA-ECMO alone, without a defined unloading and weaning strategy, has not translated into a survival benefit when applied broadly in infarct-related CS [41].

## 8. Randomized Trial Evidence: What Do DanGer Shock, ECLS-SHOCK, and ECMO-CS Add?

Much of the literature summarized above is observational, and a central limitation in this field is the relative scarcity of randomized controlled trials that test a flow-guided monitoring or management strategy, as opposed to testing a device. Three recent trials nonetheless bear indirectly on how flow parameters are used in CS. The DanGer Shock trial randomized patients with ST-elevation myocardial infarction complicated by CS to a microaxial flow pump (Impella CP) plus standard care versus standard care alone, and demonstrated a significant reduction in 180-day all-cause mortality with routine early flow-augmenting MCS, at the cost of more frequent complications including bleeding, limb ischemia, and worsening kidney function [42]. Although DanGer Shock evaluated a device rather than a monitoring strategy per se, its design and interpretation depend heavily on the flow-based physiological rationale outlined throughout this review that augmenting cardiac output in a hypoperfused patient, rather than manipulating pressure alone, is the mechanistic basis for expected benefit. The ECLS-SHOCK trial took the complementary approach of testing VA-ECMO, rather than a microaxial pump, in infarct-related CS with planned revascularization, randomizing patients to early routine extracorporeal life support plus usual medical therapy versus usual therapy alone; unlike DanGer Shock, ECLS-SHOCK found no difference in 30-day all-cause mortality between groups, and VA-ECMO was associated with higher rates of bleeding and peripheral vascular complications [41]. The contrast between DanGer Shock and ECLS-SHOCK, two trials of flow-augmenting devices in overlapping AMI-CS populations with divergent results, illustrates that device-generated flow augmentation is not uniformly beneficial and likely depends on device-specific physiology (e.g., left ventricular unloading versus afterload-increasing peripheral return), patient selection, and timing, rather than on flow augmentation as a generic mechanism. The ECMO-CS trial, which randomized patients with rapidly deteriorating or severe CS to immediate VA-ECMO implantation versus an initial conservative strategy with later escalation if needed, did not show a significant difference in the composite primary outcome, underscoring that the timing and selection criteria for flow-augmenting MCS, criteria that are themselves grounded in the hemodynamic and biomarker thresholds discussed in this review, remain an active area of investigation rather than settled practice [43]. Consistent with the terminology set out in Section 1.1, it bears emphasis that none of these three trials randomized patients to a specific measured-flow or device-flow target: device flow and blood pressure management were left largely to treating-physician discretion within broad protocol boundaries in all three trials. DanGer Shock, ECLS-SHOCK, and ECMO-CS should therefore be understood as trials of flow-augmenting device strategies, not of flow-guided monitoring strategies, a distinction that is easy to blur but is central to correctly interpreting their results in the context of this review. Taken together, these trials illustrate both the promise and the limits of flow-based physiological reasoning in guiding MCS use, and the continued need for trials that test monitoring-guided strategies as the primary intervention. DanGer Shock and ECLS-SHOCK share the same fundamental “device versus no device” trial design: a flow-augmenting device plus standard care randomized against standard care alone, in overlapping AMI-CS populations. ECMO-CS, by contrast, randomized the timing of VA-ECMO initiation (immediate versus an initial conservative strategy with rescue escalation) rather than device presence itself. Table 1 therefore places DanGer Shock and ECLS-SHOCK side by side (study design, timing of intervention, treatment strategy, primary endpoint, principal result, and key complications) as the more directly comparable evidence base, while ECMO-CS continues to be discussed above as evidence bearing on a related but conceptually distinct question of when, rather than whether, to deploy VA-ECMO.

**Table 1:** Direct comparison of study design, timing of intervention, treatment strategy, primary endpoint, and principal results in the DanGer Shock and ECLS-SHOCK trials, the two randomized “device-versus-standard-care” trials of flow-augmenting mechanical circulatory support in infarct-related cardiogenic shock.

Feature	DanGer Shock [42]	ECLS-SHOCK [41]
Study design	Multicenter, open-label RCT with blinded clinical-event adjudication	Multicenter, open-label RCT (44 centers, Germany/Slovenia)
Population	STEMI complicated by cardiogenic shock (SCAI stage C–E); planned early revascularization	AMI complicated by cardiogenic shock with planned early revascularization
Sample size	360 randomized (179 microaxial flow pump vs. 176 standard care)	420 randomized, 417 analyzed (209 ECLS vs. 208 control)
Timing of intervention	Device implanted before or immediately after primary PCI	ECLS initiated immediately after randomization, before or with revascularization
Intervention/treatment strategy	Microaxial flow pump (Impella CP) plus standard care vs. standard care alone—device vs. no device	Early VA-ECMO plus usual medical treatment vs. usual medical treatment alone—device vs. no device
Primary endpoint	All-cause mortality at 180 days	All-cause mortality at 30 days
Principal result	45.8% vs. 58.5% (HR 0.74, 95% CI 0.55–0.99, *p* = 0.04)—significant mortality reduction	47.8% vs. 49.0% (RR 0.98, 95% CI 0.80–1.19, *p* = 0.81)—no significant difference
Key complications	Composite safety endpoint 24.0% vs. 6.2% (severe bleeding, limb ischemia, hemolysis, device failure, worsening aortic regurgitation); renal-replacement therapy 41.9% vs. 26.7%	Moderate/severe bleeding 23.4% vs. 9.6% (RR 2.44); peripheral vascular complications requiring intervention 11.0% vs. 3.8% (RR 2.86)
Overall interpretation	Routine early left-ventricular-unloading flow support reduced mortality but increased vascular/bleeding and renal complications	Routine early VA-ECMO did not reduce 30-day mortality and increased bleeding/vascular complications, despite testing the same “device vs. standard care” question as DanGer Shock

## 9. Multidisciplinary Shock Teams and Protocolized Hemodynamic-Guided Care

Several observational programs have embedded routine flow-based hemodynamic assessment into structured, multidisciplinary ‘shock team’ protocols. The National Cardiogenic Shock Initiative implemented a standardized protocol emphasizing early cardiac catheterization laboratory activation, early MCS use, and routine invasive hemodynamic monitoring, and reported improved survival to discharge compared with historical single-arm data [16]. A separate single-center quality improvement initiative similarly reported improved survivorship after adopting standardized, team-based hemodynamic assessment and management, identifying elevated lactate and reduced CPO among the predictors of poor outcome that the protocol specifically targeted [17]. A contemporary review of shock team and shock center models across several North American programs concluded that hemodynamic markers such as CPO and PAPi were commonly used by these teams as explicit criteria for MCS patient selection and for assessing response to therapy, reinforcing that flow-based parameters have become embedded in real-world protocolized CS care even in the absence of a dedicated randomized trial of the monitoring strategy itself [44]. The physiological rationale underlying the use of hemodynamic monitoring specifically to guide MCS management has also been reviewed in detail elsewhere [45].

## 10. Cardiogenic Shock Heterogeneity, Phenotyping, and Evidence Gaps

Cardiogenic shock is not a single disease but a final common physiological pathway reached from etiologically and hemodynamically distinct starting points, and thresholds for the flow and perfusion parameters discussed throughout this review cannot automatically be assumed to generalize from one phenotype to another. Beyond etiology (acute myocardial infarction-related CS [AMI-CS], decompensated chronic heart failure-related CS [HF-CS], post-cardiotomy shock, fulminant myocarditis, and right ventricle-predominant shock), contemporary classification increasingly incorporates hemodynamic and clinical phenotyping. Registry-based cluster analyses from the Cardiogenic Shock Working Group have identified distinct hemodynamic phenotypes, including “non-congested”, “cardiorenal”, and “cardiometabolic” clusters, which carry different risk profiles and have since been validated in independent cardiac intensive care unit populations for predicting in-hospital and 1-year mortality [46]. Complementary work using invasive hemodynamic data has proposed classification schemes that combine cardiac power, pulmonary artery pulsatility index, and filling pressures to stratify in-hospital mortality risk beyond SCAI stage alone [47]. Right ventricle-predominant CS in particular represents a distinct phenotype (low PAPi, right atrial pressure elevated relative to pulmonary capillary wedge pressure, and disproportionate hepatic and renal congestion) that may respond poorly to LV-directed devices such as Impella alone and may instead require RV-specific or biventricular mechanical support; it is not established that CPO or PAPi thresholds validated predominantly in LV-predominant, AMI-related CS apply unchanged to HF-CS or post-cardiotomy populations, in which baseline ventricular compliance, illness chronicity, and comorbidity burden differ substantially. These etiology-specific differences are also relevant to interpreting the trial evidence in Section 8: DanGer Shock and ECLS-SHOCK both enrolled only AMI-CS with planned revascularization, so their results should not be assumed to extend unchanged to HF-CS or post-cardiotomy populations, where the underlying physiological substrate, and potentially the risk–benefit balance of a given device strategy, may differ materially. Individualizing flow and perfusion targets to hemodynamic and etiologic phenotype, rather than applying a single set of thresholds across all of CS, is therefore an important direction highlighted by, but not yet fully realized in, the parameters reviewed above.

Beyond phenotyping, several further gaps limit stronger conclusions in this field. First, essentially all of the data linking flow parameters (CPO, PAPi, LVOT VTI) to outcomes are observational; no trial to date has randomized patients to a flow-guided versus a pressure-guided (or usual care) monitoring strategy, so the extent to which measuring and acting on these parameters causally improves outcomes remains unresolved [7,10]. Second, as above, CS is etiologically heterogeneous, and thresholds derived predominantly in myocardial infarction-related CS populations, such as the original SHOCK trial registry CPO cutoffs, may not be generalizable across etiologies [14]. Third, weaning prediction models for both Impella and VA-ECMO remain limited by small sample sizes, heterogeneous definitions of ‘successful weaning’, and at best moderate discriminative accuracy, as highlighted by recent systematic reviews [33,34]. Finally, the contrast between the positive DanGer Shock result and the neutral ECLS-SHOCK and ECMO-CS results highlights that optimal patient selection and timing for flow-augmenting MCS, arguably best informed by the flow and perfusion parameters reviewed here, remains incompletely defined and is a priority for future randomized investigations [41,42,43]. From an education and quality-improvement standpoint, standardizing the timing and documentation of flow-based hemodynamic and biomarker measurements (as proposed by the SCAI Door-to-Lactate-Clearance initiative for lactate specifically [29]) may itself be a tractable, testable intervention ahead of definitive monitoring-strategy trials.

## 11. Toward an Integrated Assessment Framework

The parameters discussed throughout this review are more informative when considered together, across the trajectory of CS care, than individually. Table 2 summarizes the principal flow and perfusion parameters discussed above, alongside their physiological meaning, measurement method and typical timing, the strength of available evidence, key limitations, and their most defensible current clinical application. Parameters are explicitly characterized as supporting either a prognostic association (risk stratification) or, where the evidence permits, a more direct role in guiding a specific treatment decision, a distinction emphasized throughout this review and one we did not want to blur by tabulating these parameters without qualification. In practice, a reasonable integrated approach might combine an early, standardized biomarker panel (lactate at presentation and at a fixed early interval, per the SCAI Door-to-Lactate-Clearance concept) with SCAI stage assignment, echocardiographic LVOT VTI, and, where invasive monitoring is already in place for other reasons, CPO and PAPi, to inform the initial assessment; serial reassessment of the same parameters, tailored to the patient’s CS etiology and hemodynamic phenotype (Section 10), then supports decisions about escalation, de-escalation, and MCS weaning. We present this as a practical synthesis of the evidence reviewed above rather than as a validated or prospectively tested protocol, since, as emphasized throughout, no trial has yet tested such an integrated flow-and-perfusion-guided strategy against usual care.

**Table 2:** Summary of principal flow and perfusion parameters discussed in this review, with the primary supporting reference(s) for each parameter given in the Evidence Base column; see accompanying text for a discussion of guideline-grading limitations.

Parameter	Physiological Meaning	Measurement Method and Timing	Evidence Base	Key Limitations	Clinical Application
Cardiac output/index (PAC)	Native flow generated by the heart	Invasive thermodilution via PAC; any time PAC in place	Large observational registries (CSWG, CCCTN) [7,8,9,10,11,12]	Not randomized; confounding by indication	Prognostic (risk stratification)
Cardiac power output/index (CPO/CPI)	Combined pressure–flow “hydraulic power”	Calculated (MAP × CO/451); any time invasive data available	Strong, consistent mortality association (SHOCK registry and successors) [14,15,16,17,18,19]	No RCT of CPO-targeted therapy	Prognostic; qualitative trajectory tracking
Pulmonary artery pulsatility index (PAPi)	RV flow–pressure coupling	Calculated (PA pulse pressure/RAP); invasive	Validated across CS, HF, LVAD candidacy [22,23,24,25]	Thresholds not uniform across phenotypes	Prognostic; RV failure identification
Transpulmonary thermodilution/EVLWI	Native flow + extravascular lung water	Minimally invasive (central venous + arterial line)	Small single-center CS cohorts [26,27]	Not validated during Impella/VA-ECMO support	Investigational; feasible pre-MCS (SCAI A–C)
Lactate/lactate clearance	Global tissue hypoperfusion	Point-of-care blood test; serial (e.g., 0 and 8 h)	IABP-SHOCK II substudy; SCAI/CSWG consensus [3,4,6,28,29]	Confounded by adrenergic state, hepatic clearance, Type B causes	Prognostic; quality-metric component
CLIP composite score	Multimarker mortality risk (cystatin C, lactate, IL-6, NT-proBNP)	Laboratory panel at presentation	Single derivation cohort (AMI-CS) [30]	Not broadly externally validated	Prognostic (risk stratification)
Echocardiographic LVOT VTI	Stroke volume surrogate	Non-invasive Doppler; serial/weaning trials	Consistent predictor across weaning studies [32,33,34,35]	Doppler artefact with continuous-flow devices	Prognostic; weaning assessment
LVETc/PAWP ratio	Ejection time–filling pressure index for VA-ECMO weaning	Echo + invasive PAWP during flow-reduction trial	Single retrospective cohort [32]	Not broadly replicated	Investigational weaning parameter

Note on guideline grading: neither the 2022 SCAI SHOCK staging update [3] nor the 2025 ACC Concise Clinical Guidance on cardiogenic shock [1] assigns a formal Class-of-Recommendation/Level-of-Evidence rating to the flow and perfusion parameters summarized in Table 2; both are explicitly framed as expert-consensus or classification documents rather than graded practice guidelines. We have therefore cited the primary supporting study or studies for each parameter directly, by reference number, rather than implying a level of formal guideline endorsement that does not currently exist.

## 12. Conclusions

Blood pressure alone provides an incomplete and, in some cases, misleading picture of hemodynamic status in cardiogenic shock because compensatory vasoconstriction can preserve pressure despite a substantial reduction in flow and tissue perfusion. A substantial body of predominantly observational evidence supports the additional prognostic and, for a smaller number of parameters, management-relevant value of flow-derived hemodynamic parameters, CO and CI, CPO and CPI, and the PAPi, together with biomarkers of hypoperfusion such as lactate, lactate clearance, and composite multimarker scores. Correctly applying these parameters requires distinguishing pressure from measured and device-generated flow, and prognostic association from demonstrated treatment-guiding utility (Section 1.1, Table 2), as well as recognizing that thresholds derived largely in AMI-related CS may not transfer directly to other etiologic and hemodynamic phenotypes (Section 10). These parameters are increasingly embedded in SCAI SHOCK staging, in echocardiographic and invasive assessment protocols, and in structured algorithms for initiating, titrating, and weaning of mechanical circulatory support. The randomized evidence base, however, remains largely confined to trials of devices (DanGer Shock, ECLS-SHOCK, ECMO-CS) rather than trials of monitoring strategies themselves, and closing this gap, ideally within an individualized, phenotype-aware framework such as that outlined in Section 11, represents the most important next step for the field.

## Figures and Tables

**Figure 2:**
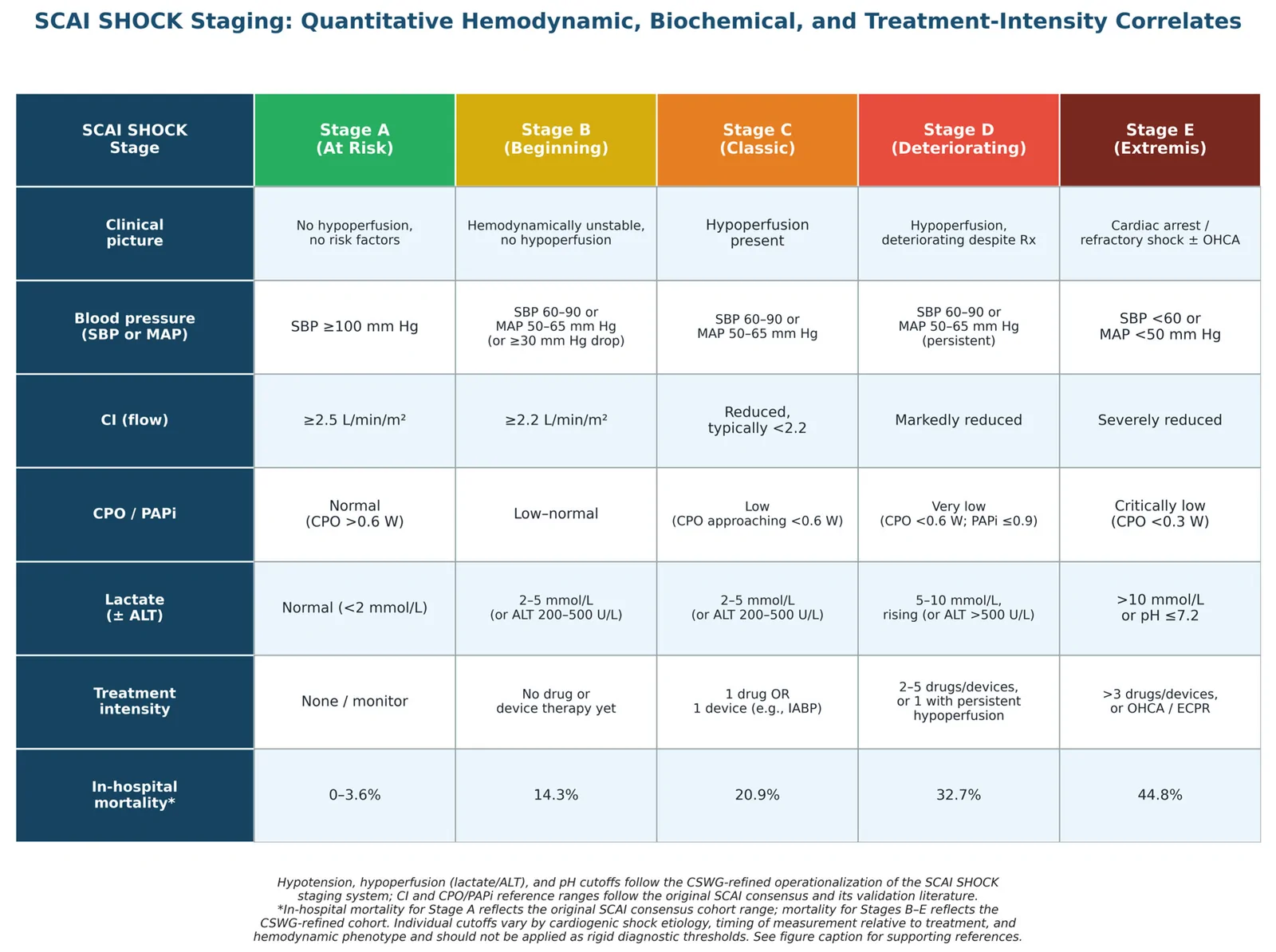
SCAI SHOCK staging with quantitative hemodynamic, biochemical, and treatment-intensity correlates across the five stages, including explicit systolic blood pressure/MAP thresholds (mm Hg), cardiac index, CPO/PAPi, lactate/ALT/pH values, treatment intensity (number of vasoactive drugs or MCS devices), and in-hospital mortality. Hypotension, hypoperfusion, and treatment-intensity cutoffs follow the CSWG-refined operationalization of the SCAI SHOCK staging system [6]; CI and CPO/PAPi reference ranges follow the original SCAI consensus and its validation literature [3,14,15,18,19,22,23,24,25]. Individual cutoffs vary by cardiogenic shock etiology, timing of measurement, and hemodynamic phenotype (Section 10) and should not be applied as rigid diagnostic thresholds.

## Data Availability

No new data were created or analyzed in this review. Data sharing is not applicable to this article.

## Abbreviations

The following abbreviations are used in this manuscript: ACC—American College of Cardiology; AMI-CS—acute myocardial infarction-related cardiogenic shock; CI—cardiac index; CLIP—cystatin C, lactate, interleukin-6, and NT-proBNP (score); CO—cardiac output; CPO—cardiac power output; CPI—cardiac power index; CS—cardiogenic shock; ECMO—extracorporeal membrane oxygenation; ECPELLA—combined VA-ECMO and Impella support; ECPR—extracorporeal cardiopulmonary resuscitation; EVLWI—extravascular lung water index; HF-CS—heart failure-related cardiogenic shock; IABP—intra-aortic balloon pump; LVEF—left ventricular ejection fraction; LVETc—corrected left ventricular ejection time; LVOT VTI—left ventricular outflow tract velocity–time integral; MAP—mean arterial pressure; MCS—mechanical circulatory support; NT-proBNP—N-terminal pro-B-type natriuretic peptide; PAC—pulmonary artery catheter; PAPi—pulmonary artery pulsatility index; PAWP—pulmonary artery wedge pressure; pVAD—percutaneous ventricular assist device; RAP—right atrial pressure; RV—right ventricle/right ventricular; SCAI—Society for Cardiovascular Angiography and Interventions; ScvO2—central venous oxygen saturation; SvO2—mixed venous oxygen saturation; SVR—systemic vascular resistance; VA-ECMO—veno-arterial extracorporeal membrane oxygenation.
